# Group painting therapy for children and adolescents with bone tumors: a quasi-experimental trial evaluating anxiety, depression, post-traumatic growth, and health-related quality of life

**DOI:** 10.3389/fpsyt.2026.1757053

**Published:** 2026-06-12

**Authors:** Jinqian Han, Mei Chan Chong, Wan Ling Lee, Enshe Jiang, Weizheng Zhang

**Affiliations:** 1 Department of Nursing Science, Faculty of Medicine, University of Malaya, Kuala Lumpur, Malaysia; 2 Institute of Nursing and Health, Henan University, Kaifeng, China; 3 Department of Nursing, Faculty of Medicine, Universiti Kebangsaan Malaysia, Kuala Lumpur, Malaysia

**Keywords:** anxiety, bone tumors, children and adolescents, depression, group painting therapy, post-traumatic growth, quality of life

## Abstract

**Background:**

Children and adolescents undergoing treatment for malignant bone tumors frequently experience heightened psychological distress, including anxiety, depression, and reduced health-related quality of life (HRQoL). Group painting therapy has been proposed as a supportive non-pharmacological strategy, yet empirical evidence in pediatric oncology remains limited. This study aimed to evaluate the impact of an eight-week structured group painting therapy program on anxiety, depression, post-traumatic growth (PTG), and HRQoL in this population.

**Methods:**

A quasi-experimental pretest–posttest study was conducted among children and adolescents (aged 8–18 years) with primary malignant bone tumors at Henan Cancer Hospital from December 2023 to December 2024. A total of 76 participants were consecutively enrolled and allocated to an intervention group receiving group painting therapy (n=39) or a control group receiving standard nursing care (n=37) using a nonrandomized ward-based approach. Anxiety, depressive symptoms, PTG, and HRQoL were assessed at baseline and post-intervention using validated instruments, including SCARED, DSRSC, PTGI, and PedsQL™ 3.0 Cancer Module.

**Results:**

Compared with the control group, the intervention group demonstrated greater reductions in anxiety and depressive symptoms, along with significant improvements in PTG and selected HRQoL domains. Notable improvements were observed in emotional functioning, social communication, generalized anxiety, somatic symptoms, personal strength, and appreciation of life. No significant between-group differences were observed in physical symptom domains such as pain and nausea.

**Conclusions:**

Group painting therapy was associated with improvements in emotional well-being, PTG, and selected HRQoL domains in children and adolescents with primary bone tumors. These findings suggest that painting therapy may be a promising adjunct to routine pediatric oncology care for promoting resilience and psychological recovery.

## 1 Introduction

Bone tumors are rare but aggressive neoplasms that pose a serious threat to the physical and psychological health of children and adolescents (1). Osteosarcoma and Ewing sarcoma are the most common malignant bone tumors in this age group and often present during puberty, a period marked by rapid skeletal growth and significant psychosocial development (2, 3). Although advances in multidisciplinary treatment have improved survival, many patients continue to experience prolonged treatment courses involving surgery, chemotherapy, and rehabilitation, and a considerable proportion still present with metastatic disease at diagnosis (4). Extended hospitalizations, repeated invasive procedures, and functional impairments disrupt age-appropriate developmental tasks and increase emotional stress, making bone tumors a major pediatric health concern (5).

Beyond physical morbidity, children and adolescents with bone tumors face substantial psychological challenges throughout the disease trajectory. Repeated medical procedures, uncertainty regarding prognosis, loss of physical independence, and treatment-related body changes contribute to heightened emotional vulnerability (6). Anxiety and depressive symptoms are among the most frequently reported psychological problems in this population, with prevalence rates ranging from 25% to 35%, and are particularly pronounced during early treatment and periods of functional decline (7). Adolescents may be especially affected, as limb-salvage surgery, amputation, or visible treatment-related changes can negatively influence self-esteem, peer relationships, and identity development (8). Psychological distress in pediatric oncology has been associated with poorer treatment adherence, reduced health-related quality of life (HRQoL), and longer-term psychosocial difficulties (9). Despite increasing recognition of distress, systematic psychosocial interventions in inpatient oncology settings remain limited, and timely support is often constrained by staffing and resource availability.

In addition to psychological distress, some young patients demonstrate positive psychological changes after experiencing cancer, commonly termed post-traumatic growth (PTG). PTG refers to an increased appreciation for life, stronger interpersonal connections, greater personal resilience, and the discovery of new opportunities that emerge after facing significant adversity (10). While PTG has been widely studied in adult cancer survivors, fewer investigations have examined PTG in pediatric bone tumor populations, and even fewer have evaluated whether structured psychosocial interventions can actively promote PTG in this group (11). As children and adolescents are still in the process of forming core beliefs and identity, their PTG experiences may differ substantially from those of adults (12). Understanding how targeted interventions influence PTG is therefore essential for designing developmentally appropriate supportive care strategies.

Health-related quality of life (HRQoL) is another critical outcome for pediatric bone tumor patients. Long treatment duration, frequent hospitalizations, pain, fatigue, and disruptions in school and social life can significantly impair physical, emotional, and social functioning (13). Psychological distress has been consistently linked to poorer HRQoL in pediatric oncology populations and may persist beyond treatment completion (14). Among childhood cancer survivors, individuals treated for bone tumors often report one of the highest burdens of long-term physical and psychosocial sequelae, underscoring the importance of early psychosocial support aimed at improving HRQoL trajectories.

Despite growing awareness of psychosocial challenges, supportive care for children and adolescents with bone tumors remains fragmented and is often adapted from adult oncology models (15). In busy inpatient settings, limited access to specialized mental health professionals and time constraints may restrict the delivery of systematic, developmentally tailored interventions (16). As a result, there is a clear need for feasible, ward-based psychosocial programs that can be integrated into routine care.

In recent years, increasing attention has been directed toward non-pharmacological interventions for alleviating psychological distress in patients with cancer. These approaches include cognitive-behavioral therapy, mindfulness-based interventions, psychosocial support, and art-based therapies, which have been shown to help reduce anxiety and depressive symptoms (17). Such interventions are generally safe, non-invasive, and well accepted by patients (18, 19). Among these approaches, expressive arts therapy has been increasingly applied as a complementary strategy in clinical practice. It enables patients to externalize internal emotions, thereby reducing psychological distress. Through creative activities patients are able to express feelings that may be difficult to articulate verbally (20). Compared to interventions that rely primarily on verbal communication, painting-based approaches may be particularly suitable for children and adolescents who struggle to express their emotions verbally. Painting therapy, in particular, facilitates emotional exploration, enhances self-awareness, and promotes emotional relief in a non-threatening way (21). Group-based interventions also draw on principles of group dynamics, where shared experiences, peer modeling and mutual support promote emotional adjustment and reduce feelings of isolation (22, 23). Previous studies have also suggests that art-based interventions may alleviate anxiety and depressive symptoms among pediatric oncology patients (24).

From a theoretical perspective, structured group painting therapy may influence psychological outcomes through multiple pathways, including emotional expression and regulation, cognitive reframing of illness experiences, enhancement of perceived social support, and meaning-making processes that contribute to PTG (25). Nevertheless, existing evidence remains limited. Most prior studies have examined mixed pediatric cancer populations, outpatient settings, or individual art-based interventions and have been characterized by methodological heterogeneity and small sample sizes (26). Moreover, little research has specifically examined structured group-based painting therapy in hospitalized children and adolescents with malignant bone tumors, particularly with the simultaneous assessment of anxiety, depression, post-traumatic growth, and tumor-specific HRQoL. Addressing these gaps is important for developing developmentally appropriate and context-sensitive psychosocial support strategies in inpatient oncology settings. Therefore, this quasi-experimental study was designed to evaluate the effects of an eight-week structured group painting therapy program on anxiety, depressive symptoms, post-traumatic growth, and quality of life among hospitalized children and adolescents with malignant bone tumors.

## 2 Methods

### 2.1 Study design and setting

This study employed a two-arm quasi-experimental design with a non-equivalent control group and pretest–posttest assessments to evaluate the effectiveness of group painting therapy in children and adolescents with bone tumors. The study was conducted in the Department of Bone and Soft Tissue Oncology at Henan Cancer Hospital, a provincial referral center for musculoskeletal malignancies. The department includes two comparable bone tumor inpatient wards, each with similar bed capacity, staffing, and patient case mix.

Due to ward management policies and routine bed-allocation procedures, individual randomization was not feasible. Admission to the two wards was determined primarily by bed availability and treatment scheduling rather than by patients’ clinical characteristics. Patients in Ward 2 were assigned to the intervention group, and patients in Ward 1 constituted the control group. Patients generally remained under the same medical team throughout their inpatient treatment, minimizing cross-group contamination.

Because allocation occurred at the ward level, no intraclass correlation coefficient (ICC) was estimated and no design effect adjustment was applied in the sample size calculation or statistical analyses. All outcomes were assessed by trained research assistants who were independent of the intervention team and blinded to group allocation. Data collectors received coded participant identifiers without group labels and followed standardized administration procedures. Baseline demographic and clinical characteristics were compared between groups to assess potential imbalances resulting from the non-randomized allocation.

### 2.2 Participants

Participants were recruited from the two inpatient wards of the Department of Bone and Soft Tissue Oncology between December 2023 and December 2024. Recruitment was limited to hospitalized patients. All potentially eligible patients during the study period were consecutively screened by trained research nurses using electronic medical records and daily admission lists. Reasons for refusal were documented.

Eligibility criteria included:

(1) age 8–18 years;
(2) diagnosis of a malignant bone tumor;
(3) medical stability and ability to engage in group-based activities;
(4) adequate communication ability; and
(5) currently receiving or having recently completed standard treatment (surgery, chemotherapy, or radiotherapy).

Exclusion criteria were: (1) severe cognitive impairment or inability to communicate; (2) unstable medical condition requiring intensive care or restricting physical activity; (3) concurrent participation in other psychosocial interventions; (4) diagnosis of severe pre-existing psychiatric disorders unrelated to cancer.

### 2.3 Sample size

The sample size was calculated based on detecting a clinically meaningful difference in the primary outcome (total SCARED score) between two independent groups using the following formula:


$$n\;=\;\frac{2\;{\left(Z_{a/2}\;+\;Z_{\beta }\right)}^{2}\sigma^{2}}{\delta^{2}}$$

Where $Z_{\alpha }/2$=1.96 for a two-tailed test with α=0.05, and $Z_{\beta }$=1.28 for 90% power (β=0.10). The standard deviation was estimated at (σ=6.85) and the expected mean difference (δ=5.5) for the primary outcome (total SCARED score) were derived from a prior pediatric oncology study that reported anxiety variability and clinically meaningful change thresholds (27). Substituting these values into the formula yielded a required sample size of 33 participants per group. Accounting for an expected 20% attrition rate, the final planned enrollment was 42 participants per group, yielding a total of 84 participants.

### 2.4 Study procedure and data collection

Eligible patients were systematically screened from the two inpatient wards of the Department of Bone and Soft Tissue Oncology at Henan Cancer Hospital. After confirming eligibility, written informed consent was obtained from both the participant and their legal guardian, along with assent from children when appropriate. Participants were assigned according to their ward (Ward 2: intervention group; Ward 1: control group).

Baseline assessments were completed prior to intervention initiation, and post-intervention assessments were conducted within 24 hours of the final session. All outcome measures were administered by blinded research assistants not involved in intervention delivery. Attendance and protocol deviations were documented throughout the study. Analyses were conducted using a per-protocol approach, including participants who completed both baseline and post-intervention assessments. An intention-to-treat analysis was not performed because participants who withdrew did not complete post-intervention assessments, resulting in missing post-intervention outcome data.

To enhance adherence, consistent communication with caregivers was facilitated through a dedicated WeChat group, which served as a channel for reminders and logistical assistance. Emotional distress was monitored throughout participation, and referrals to psychological services were made when clinically indicated. Upon completion, participants received a set of picture books as a token of appreciation for their participation.

### 2.5 Intervention

#### 2.5.1 Intervention group

Participants assigned to the intervention group received standard routine care plus an eight-week structured group painting therapy program delivered in the inpatient setting. The intervention was developed by a multidisciplinary team including the principal investigator, an orthopedic oncology physician, senior oncology nurses, a certified art therapy specialist, and master-level nurses. Two oncology nurses who completed standardized competency-based training in painting therapy served as facilitators, with ongoing supervision provided by a certified art therapist to ensure fidelity and clinical quality. The intervention program was informed by Expressive Art Therapy Theory and Lewin’s Group Dynamics Theory, emphasizing creative expression for emotional processing and peer interaction for therapeutic engagement (28, 29). Prior to implementation, all intervention team members completed systematic training covering expressive art therapy principles, pediatric psychosocial oncology, group facilitation skills, standardized procedures, and ethical practice. Additional training workshops were delivered by external experts in pediatric psychology and art therapy. The intervention protocol underwent expert review by ten senior specialists in pediatric oncology, psychology, nursing, and expressive arts therapy (≥10 years of experience) and was revised based on their feedback to enhance feasibility, psychological safety, and developmental appropriateness. The finalized intervention protocol consisted of eight consecutive weekly group meetings, each lasting 60–90 minutes. Considering the fluctuating clinical conditions and treatment-related symptoms in pediatric oncology patients, the meeting schedule was coordinated with the clinical team and implemented flexibly, always prioritizing patient safety. Sessions were scheduled to avoid periods of treatment-related discomfort and conducted in a dedicated child-friendly room. Sessions were arranged to avoid periods of severe pain, nausea, chemotherapy-related toxicity, radiotherapy-related discomfort, or other treatment-related symptoms, and were conducted in a dedicated child-friendly room. If a participant was unable to attend a scheduled meeting for medical reasons, attendance was not mandatory; absences were recorded, and the participant could continue to attend subsequent meetings once their clinical condition stabilized. Participants who completed baseline and post-intervention assessments and attended at least six of the eight meetings were included in the compliance analysis. Group sizes were 5–7 people to promote participation and meaningful interaction.

Each session followed a standardized three-step structure: (1) Warm-up: brief activity to support emotional readiness and group cohesion. (2) Thematic painting task: guided expressive activity based on themes such as emotions, coping, illness experience, identity, or strengths. (3) Sharing and reflection: voluntary group discussion supporting meaning-making and peer support. The intervention focused on emotional expression rather than artistic skill, and no evaluation of drawing performance was conducted. A standardized intervention manual guided session delivery. Facilitators documented attendance, session content, participant engagement, and key observations after each session. All art materials were non-toxic, single-use, and compliant with hospital infection-control protocols for immunocompromised children. Participants’ emotional responses were continuously monitored, and those exhibiting acute psychological distress were provided immediate support and referred to mental health services when necessary. All artwork remained the property of the participant and was not displayed or shared without explicit permission. A summary of the intervention structure is provided in **Table 1**.

**Table 1:** Structure and content of the group painting therapy program.

Session	Theme	Ice-breaking activity	Therapeutic goal
1	Building Connections	Passing the Flower	Establish group cohesion and enhance self-awareness through reflective creative expression.
2	Exploring Inner Emotions	Mood relay & Emotion masks	Facilitate recognition and release of suppressed emotions to reduce distress.
3	Symbolic Self-Reflection	“Big and Small Watermelon” game	Promote understanding of personal strengths and support systems through symbolic imagery.
4	Creating Safe Emotional Spaces	Paper Lotus folding	Encourage emotional safety, acceptance, and expression of inner experiences.
5	Visualizing Hope and Affection	Wishing Lantern	Transform hopes and affectionate feelings into concrete visual forms to foster optimism.
6	Reconstructing Positive Self-Image	Sharing favorite poem/Chengyu	Support positive self-identity reconstruction via projection and imagination.
7	Future Aspirations	“Silk Road of Dreams” activity	Promote future-oriented thinking and meaning reconstruction.
8	Illness Reframing and Coping	Storytelling: Fighting the Monster	Facilitate illness reframing, strengthen resilience, and build adaptive coping strategies.

#### 2.5.2 Control group

The control group received the standard routine care according to established ward nursing protocols. Routine care included environmental infection control (air purification and disinfection), health education related to diagnosis and treatment, monitoring of vital signs, nutritional support, skin care, and postoperative or chemotherapy-related care. Basic psychological support such as reassurance, distraction strategies during procedures, and emotional encouragement was provided by nursing staff as needed.

In addition, participants attended a weekly 40-minute nurse-facilitated parent–child peer meeting. These sessions focused on discussion of common concerns, experience sharing, and social interaction, without structured psychological guidance, expressive-art activities, or other therapeutic elements. This served as an active attention control, providing social engagement comparable in time to the intervention but without structured psychological guidance, expressive-art activities, or other therapeutic elements. In this study, the symptom management protocols were consistent across all groups. Both groups used analgesics and antiemetics according to the same institutional protocols and individual clinical needs, and the painting intervention did not replace, limit, or alter these treatments.

### 2.6 Research instruments

The primary outcome was total anxiety score measured using the Screen for Child Anxiety Related Emotional Disorders (SCARED). Secondary outcomes included depressive symptoms (DSRSC), post-traumatic growth (PTGI), and health-related quality of life (PedsQL™ 3.0).

#### 2.6.1 Sociodemographic and clinical questionnaire

Sociodemographic and clinical characteristics were collected using a self-designed questionnaire. Items included age, sex, education level, family structure, household income, tumor pathological type, treatment modality, tumor site, presence of metastasis, and amputation status.

#### 2.6.2 Screen for child anxiety related emotional disorders

Anxiety symptoms were evaluated using the 41-item SCARED, which assesses five domains: generalized anxiety, separation anxiety, social phobia, somatic symptoms, and school avoidance (30). Each item is scored from 0 to 2 according to symptom frequency over the previous three months. Higher total and subscale scores indicate greater severity of anxiety symptoms. The Chinese adaptation shows acceptable reliability and diagnostic performance, with Cronbach’s α ranging from 0.43 to 0.89 (31).

#### 2.6.3 Depression self-rating scale for children

Depressive symptoms were measured with the 18-item DSRSC, where items are rated on a 0–2 scale. Higher total scores reflect greater depressive severity, and a cutoff of 15 is commonly used to indicate probable depression (32). The Chinese version demonstrates good psychometric quality in child populations, including a Cronbach’s α of 0.73 and diagnostic sensitivity and specificity of 0.86 and 0.82, respectively (33).

#### 2.6.4 Post-traumatic crowth inventory

Post-traumatic growth was assessed using a 21-item modified Chinese version of the PTGI (34). Items are scored on a 6-point Likert scale and cover five dimensions: Relating to Others, New Possibilities, Personal Strength, Spiritual Change, and Appreciation of Life. Higher scores reflect greater perceived positive psychological changes following traumatic experiences. This version shows excellent internal consistency (Cronbach’s α = 0.926) and strong construct validity, making it suitable for assessing positive psychological changes following major illness (34).

#### 2.6.5 Pediatric quality of life inventory™ 3.0 cancer module

Health-related quality of life was assessed using the child self-report version of the PedsQL™ 3.0 Cancer Module, comprising 27 items across eight domains: pain and hurt, nausea, procedural anxiety, treatment anxiety, worry, cognitive problems, perceived physical appearance, and communication (35). Responses are scored on a five-point Likert scale and transformed to 0–100 scores, with higher values indicating better QoL. The Chinese version has demonstrated strong internal reliability (Cronbach’s α=0.84–0.99) and solid structural validity in pediatric oncology samples (36).

### 2.7 Data analysis

Data analysis was performed using IBM SPSS Statistics 26.0. Descriptive statistics summarized demographic and clinical characteristics. Normality was assessed using Q–Q plots and the Shapiro–Wilk test. Normally distributed variables were presented as mean ± standard deviation (SD) and analyzed using independent-samples t-tests (between-group) and paired t-tests (within-group). Non-normally distributed variables were reported as median with interquartile range (IQR) and analyzed using the Mann–Whitney U test for between-group comparisons and the Wilcoxon signed-rank test for within-group comparisons. For non-normally distributed outcomes, bias-corrected and accelerated (BCa) bootstrap procedures with 1, 000 resamples were used to estimate 95% confidence intervals (CIs) for change scores and between-group differences in change. Categorical variables were expressed as frequencies and percentages and compared using the chi-square test or Fisher’s exact test, as appropriate. Effect sizes were calculated as Cohen’s *d* for parametric comparisons and as rank-biserial correlation (*r*), derived from the standardized *Z* statistic, for non-parametric comparisons. Between-group differences in change scores were calculated and reported with corresponding 95% confidence intervals. All statistical tests were two-sided, and Bonferroni correction was applied within each outcome family to control for multiple comparisons, with statistical significance interpreted based on the adjusted *p* values.

## 3 Results

### 3.1 Participant flow

**Figure 1:**
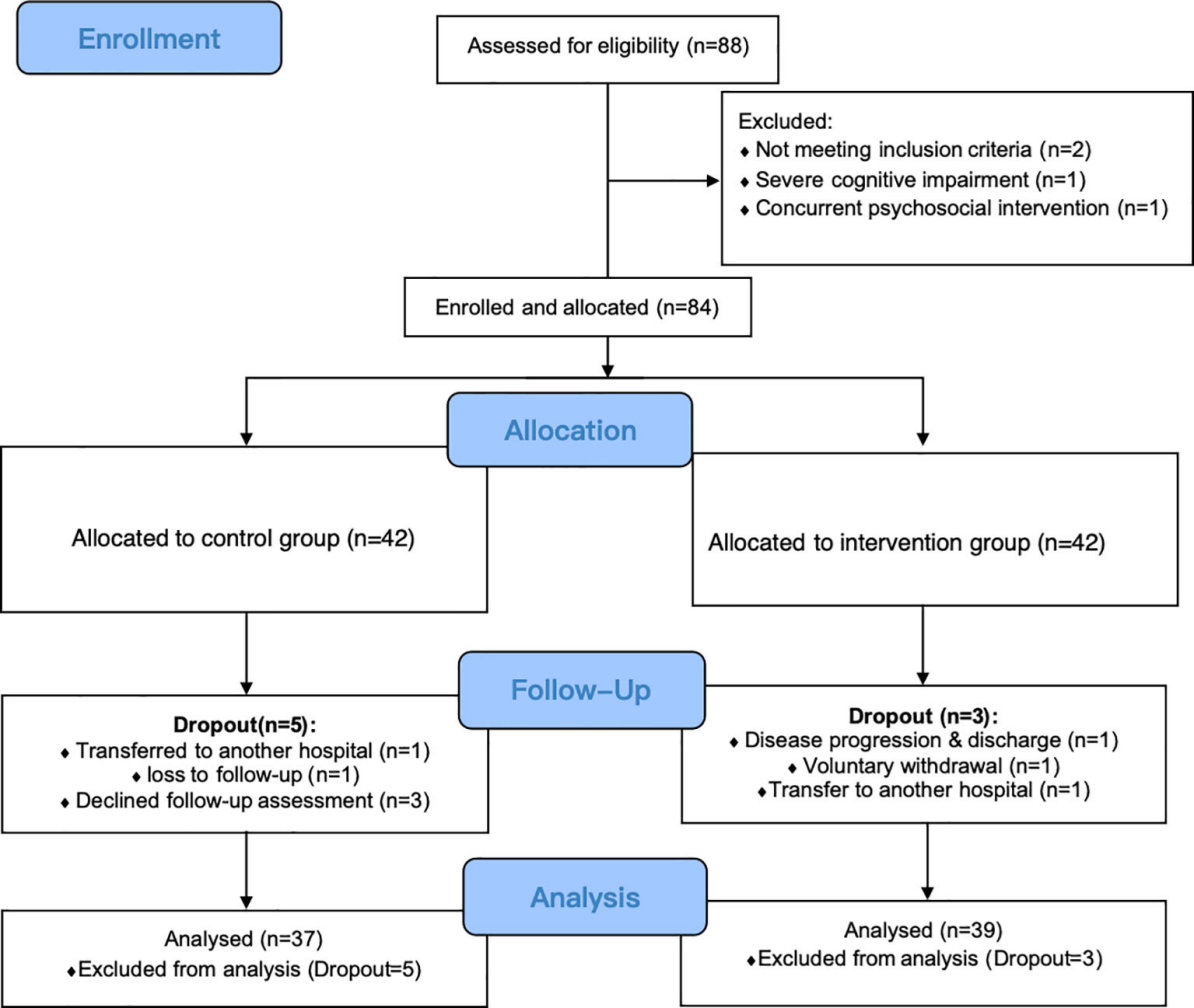
Enrollment flow diagram.

A total of 88 pediatric bone tumor patients were screened for eligibility. Four patients were excluded (age<8 years, n=2; severe cognitive impairment, n=1; concurrent psychosocial intervention, n=1). The remaining 84 participants were enrolled and allocated according to ward assignment (intervention group, n = 42; control group, n = 42). During the study period, three participants in the intervention group withdrew due to disease progression (n=1), voluntary withdrawal (n=1), or transfer to another hospital (n=1). In the control group, five participants were lost owing to hospital transfer (n=2), loss to follow-up (n=1), or refusal to complete the final assessment (n=2). None of the withdrawals were related to the intervention. The overall attrition rate was 9.52% (8/84). A per-protocol analysis was conducted including 76 participants (intervention n=39; control n=37) (**Figure 1**). No imputation procedures were applied for missing outcome data. Participants without post-intervention assessments were excluded from outcome analyses.

### 3.2 Intervention exposure and fidelity

Among participants in the intervention group who completed follow-up (n = 39), eight sessions were delivered as planned. The mean session duration was 81.1 minutes (SD = 6.3; range, 70–90 minutes). The average group size across sessions was 5.6 participants (range, 5–7). The overall attendance rate across sessions was 93.8%. The median number of sessions attended per participant was 8 (range, 6–8). Missed sessions was recorded, and participants were allowed to attend subsequent classes once their physical condition stabilizes. No session was interrupted due to acute medical discomfort, and no intervention-related adverse events or serious psychological distress requiring discontinuation of treatment were observed.

### 3.3 Baseline demographic, clinical, and psychological characteristics

Baseline demographic, clinical, and psychological characteristics were comparable between groups (**Table 2**). No statistically significant differences were observed in age, gender, tumor type, distant metastasis, treatment modality, household income, insurance type, affected anatomical site, amputation status, or baseline outcome measures (all *p*>0.05). Age showed a borderline but non-significant difference between groups (*p* = 0.051). All outcome analyses were based on the per-protocol sample (n=76), and denominators were consistent across outcome tables.

**Table 2:** Baseline sociodemographic, clinical, and psychological characteristics of patients.

Characteristic	Intervention group (n = 39)	Control group (n = 37)	Test statistic	*P*-value
Gender			χ²=0.915	0.339
Male	20 (51.3%)	23 (62.2%)		
Female	19 (48.7%)	14 (37.8%)		
Age (years)	12.77 ± 2.75	13.95 ± 2.39	*t* = 1.983	0.051
Household Income (CNY/month)			χ²=0.954	0.329
< 5000	21 (53.8%)	24 (64.9%)		
≥ 5000	18 (46.2%)	13 (35.1%)		
Type of Health Insurance			χ²=0.005	0.946
Medical insurance	36 (92.3%)	34 (91.9%)		
Self-pay	3 (7.7%)	3 (8.1%)		
Presence of Distant Metastasis			χ²=0.014	0.906
Yes	8 (20.5%)	8 (21.6%)		
No	31 (79.5%)	29 (78.4%)		
Diagnosis Type			χ²=0.321	0.956
Osteosarcoma	17 (43.6%)	16 (43.2%)		
Ewing’s Sarcoma	15 (38.5%)	13 (35.1%)		
Chondrosarcoma	2 (5.1%)	3 (8.1%)		
Others	5 (12.8%)	5 (13.5%)		
Affected Area			χ²=0.781	0.854
Femur	26 (66.7%)	22 (59.5%)		
Tibia	7 (17.9%)	7 (18.9%)		
Humerus	3 (7.7%)	5 (13.5%)		
Others	3 (7.7%)	3 (8.1%)		
Amputation			χ²=0.006	0.937
Yes	4 (10.3%)	4 (10.8%)		
No	35 (89.7%)	33 (89.2%)		
Treatment Modality			χ²=0.233	0.972
Surgery + Chemotherapy	27 (69.2%)	27 (73.0%)		
Chemotherapy	8 (20.5%)	6 (16.2%)		
Surgery	2 (5.1%)	2 (5.4%)		
Others	2 (5.1%)	2 (5.4%)		
Family History			χ²=0.003	0.957
Yes	2 (5.1%)	2 (5.4%)		
No	37 (94.9%)	35 (94.6%)		
Only-child Status			χ²=0.158	0.691
Yes	9 (23.1%)	10 (27.0%)		
No	30 (76.9%)	27 (73.0%)		
Educational Level			χ²=3.523	0.172
Primary School	12 (30.8%)	5 (13.5%)		
Junior High School	21 (53.8%)	23 (62.2%)		
Senior High School	6 (15.4%)	9 (24.3%)		
DSRSC (baseline)	14.10 ± 0.88	14.54 ± 1.37	*t* = -1.669	0.099
SCARED (baseline)	25.0 [24.0, 26.0]	25.0 [23.0, 26.0]	Z=-0.327	0.743
PTGI (baseline)	48.36 ± 3.52	49.32 ± 4.59	*t* = -1.031	0.306
PedsQL (baseline)	48.15 [40.74, 50.93]	49.07 [48.15, 50.00]	Z=-1.014	0.311

Continuous variables are presented as mean ± SD or median [IQR] as appropriate. Categorical variables are presented as n (%). p values represent between-group comparisons.

### 3.4 Study outcomes

#### 3.4.1 Depression and anxiety outcomes

After eight weeks, depressive symptoms decreased significantly in both groups. The intervention group showed a greater reduction (Δ=−1.38, 95%CI −1.69 to −1.08) compared with the control group (Δ=−0.73, 95%CI −1.10 to −0.35). The between-group difference in change scores was statistically significant (ΔΔ=−0.65, 95%CI −1.12 to −0.15), with a moderate effect size (*d* = 0.61).

For anxiety, the intervention group demonstrated a substantial reduction in total SCARED score (Δ= −8.69, 95%CI −9.27 to −8.15), whereas the control group showed a smaller decrease (Δ=−2.35, 95%CI −2.87 to −1.84). The between-group difference was significant (ΔΔ=−6.34, 95%CI −7.09 to −5.58), with a large effect size (*r* = 0.86).

At the subscale level, anxiety reductions were more pronounced in the intervention group than in the control group. Between-group comparisons indicated significantly greater improvements in the intervention group across all anxiety domains, with effect sizes ranging from *r* = 0.52 to 0.82 (**Table 3**).

**Table 3:** Changes in anxiety and depression scores from baseline to post-intervention.

Outcome	Group	Baseline	Post-intervention	Within group comparison	Δ (95% CI)	ΔΔ (95% CI)	Effect size	*p*	Adj. *p*
Total SCARED score	Intervention group(n=39)	25.0 [24.0, 26.0]	16.0 [15.0, 17.0]	Z=-5.477, *p*< 0.001	−8.69 (−9.27, −8.15)	-6.34 (-7.09, -5.58)	*r* = 0.86	< 0.001	< 0.006
	Control group(n=37)	25.0 [23.0, 26.0]	22.0 [21.0, 24.0]	Z =-4.836, *p*< 0.001	−2.35 (−2.87, −1.84)				
Somatic symptoms	Intervention group(n=39)	7.0 [7.0, 8.0]	5.00 [4.00, 5.00]	Z=-5.507, *p*< 0.001	−2.44 (−2.83, −2.08)	-2.17(-2.59, -1.73)	*r* = 0.82	< 0.001	< 0.006
	Control group(n=37)	7.0 [7.0, 7.0]	7.0 [6.5, 7.0]	Z=-2.236, *p* = 0.025	−0.27 (−0.50, −0.05)				
Generalized anxiety	Intervention group(n=39)	5.0 [5.0, 5.0]	4.0 [3.0, 4.0]	Z=-5.249, *p*< 0.001	−1.31 (−1.54, −1.07)	-1.12 (-1.52, -0.76)	*r* = 0.59	< 0.001	< 0.006
	Control group(n=37)	5.0 [4.5, 5.0]	5.0 [4.0, 5.0]	Z=-1.366, *p* = 0.172	−0.19 (−0.42, 0.06)				
Separation anxiety	Intervention group(n=39)	4.0 [4.0, 4.0]	3.00 [3.00, 3.00]	Z=-5.514, *p*< 0.001	−1.08 (−1.24, −0.91)	-0.83 (-1.07, -0.60)	*r* = 0.65	< 0.001	< 0.006
	Control group(n=37)	4.0 [4.0, 5.0]	4.0 [4.0, 4.0]	Z=-2.714, *p* = 0.007	−0.24 (−0.41, −0.10)				
Social phobia	Intervention group(n=39)	5.0 [5.0, 7.0]	4.0 [4.0, 4.0]	Z=-5.512, *p*< 0.001	−2.00 (−2.28, −1.73)	-1.32 (-1.83, -0.84)	*r* = 0.57	< 0.001	< 0.006
	Control group(n=37)	6.0 [5.0, 7.0]	5.0 [4.5, 7.0]	Z=-3.081, *p* = 0.002	−0.68 (−1.03, −0.32)				
School avoidance	Intervention group(n=39)	3.0 [2.0, 3.0]	1.0 [1.0, 1.0]	Z=-5.453, *p*< 0.001	−1.87 (−2.11, −1.62)	-0.90(-1.26, -0.57)	*r* = 0.52	< 0.001	<0.006
	Control group(n=37)	3.0 [2.0, 3.0]	2.0 [1.0, 2.0]	Z=-4.627, *p*< 0.001	−0.97 (−1.23, −0.71)				
Total DSRSC score	Intervention group(n=39)	14.10 ± 0.88	12.72 ± 0.60	*t* = 9.247, *p*< 0.001	-1.38(−1.69, −1.08)	−0.65(-1.12, -0.15)	*d* = 0.61	0.010	0.010
	Control group(n=37)	14.54 ± 1.37	13.81 ± 1.12	*t* = 3.648, *p*< 0.001	−0.73 (−1.10, −0.35)				

SCARED, Screen for Child Anxiety Related Emotional Disorders; DSRSC, Depression Self-Rating Scale for Children. Values are mean ± SD or median [IQR]; Δ, within-group change; ΔΔ, between-group difference in change. Effect sizes are Cohen’s *d* or rank-biserial *r*; *p* values represent between-group comparisons; Adj. *p*, Bonferroni-adjusted *p* value for between-group change.

#### 3.4.2 Post-traumatic growth and health-related quality of life

Total PTGI scores increased significantly in both groups. The intervention group demonstrated a greater increase (Δ=16.44, 95%CI 15.20 to 17.68) compared with the control group (Δ= 9.92, 95%CI 8.97 to 10.87). The between-group difference in change was statistically significant (ΔΔ=6.52, 95%CI 4.72 to 8.31, *p*< 0.001), with a moderate effect size (*d* = 0.68).

At the domain level, between-group differences favored the intervention for Relating to Others (*r* = 0.35), New Possibilities(*r* = 0.32), Personal Strength (*r* = 0.57), and Appreciation of Life (*r* = 0.38). No significant between-group difference was observed for Spiritual Change (*p* = 0.331) (**Table 4**).

**Table 4:** Changes in post-traumatic growth from baseline to post-intervention.

Outcome	Group	Baseline	Post-intervention	Within-group comparison	Δ (95% CI)	ΔΔ (95% CI)	Effect size	*p*	Adj. *p*
PTGI Total score	Intervention group(n=39)	48.35 ± 3.52	64.79 ± 4.46	*t* = -23.922, *p*< 0.001	16.44 (15.20, 17.68)	6.52 (4.72, 8.31)	*d* = 0.68	<0.001	< 0.006
	Control group(n=37)	49.32 ± 4.59	59.24 ± 4.69	*t* = -17.204, *p*< 0.001	9.92 (8.97, 10.87)				
Relating to others	Intervention group(n=39)	16.0 [16.0, 18.0]	22.0 [19.0, 25.0]	Z=-5.190, *p*< 0.001	4.54 (3.22, 5.86)	2.67 (1.32, 4.02)	*r* = 0.35	0.002	0.012
	Control group(n=37)	16.0 [15.0, 22.0]	19.0 [17.0, 25.0]	Z=-3.990, *p*< 0.001	1.86 (1.06, 2.67)				
New Possibilities	Intervention group(n=39)	10.0 [9.0, 12.5]	13.0 [11.0, 15.0]	Z=-5.374, *p*< 0.001	2.49 (2.07, 2.90)	0.75 (0.13, 1.36)	*r* = 0.32	0.005	0.030
	Control group(n=37)	10.0 [8.0, 13.0]	12.0 [11.0, 14.5]	Z=-4.938, *p*< 0.001	1.73 (1.24, 2.22)				
Personal Strength	Intervention group(n=39)	9.0 [8.0, 9.0]	12.0 [11.0, 14.0]	Z=-5.327, *p*< 0.001	3.90 (3.28, 4.51)	1.95 (1.23, 2.65)	*r* = 0.57	<0.001	<0.006
	Control group(n=37)	9.0 [8.0, 9.0]	10.0 [10.0, 12.0]	Z=-5.236, *p*< 0.001	1.95 (1.55, 2.35)				
Spiritual Change	Intervention group(n=39)	4.0 [4.0, 5.0]	6.0 [6.0, 7.0]	Z=-5.258, *p*< 0.001	1.87 (1.51, 2.23)	0.22 (−0.16, 0.61)	*r* = 0.11	0.331	1.000
	Control group(n=37)	4.0 [4.0, 4.0]	6.0 [5.5, 6.0]	Z=-5.479, *p*< 0.001	1.65 (1.47, 1.83)				
Appreciation of Life	Intervention group(n=39)	7.0 [7.0, 8.0]	11.0 [10.0, 11.0]	Z=-5.498, *p*< 0.001	3.64 (3.30, 3.99)	0.91 (0.45, 1.38)	*r* = 0.38	0.001	0.006
	Control group(n=37)	7.0 [7.0, 8.0]	10.0 [10.0, 11.0]	Z=-5.222, *p*< 0.001	2.73 (2.36, 3.10)				

PTGI, Posttraumatic Growth Inventory. Values are mean ± SD or median [IQR]; Δ, within-group change; ΔΔ, between-group difference in change. Effect sizes are Cohen’s d or rank-biserial *r*; *p* values represent between-group comparisons; Adj. *p*, Bonferroni-adjusted p value for between-group change.

Total PedsQL™ 3.0 scores improved significantly in both groups, with a greater increase in the intervention group (Δ=16.33, 95%CI 13.43 to 19.65) compared with the control group (Δ=8.91, 95%CI 6.34 to 12.19). The between-group difference in change was statistically significant (ΔΔ= 7.43, 95%CI 3.23 to 11.62, *p*< 0.001), with a moderate effect size (*r* = 0.42).

After Bonferroni correction, significant between-group differences remained for treatment anxiety (*r* = 0.34), procedural anxiety (*r* = 0.46), cognitive problems (*r* = 0.34), perceived physical appearance (*r* = 0.33), and communication (*r* = 0.35). The between-group difference for worry did not remain statistically significant after Bonferroni correction. No significant between-group differences were found for pain and hurt or nausea (**Table 5**).

**Table 5:** Changes in health-related quality of life from baseline to post-intervention.

Outcome	Group	Baseline	Post-intervention	Within-group comparison	Δ (95% CI)	ΔΔ (95% CI)	Effect size	*p*	Adj. *p*
Total PedsQL™ 3.0 score	Intervention group(n=39)	48.15 [40.74, 50.93]	66.66 [56.48, 67.59]	Z=-5.375, *p*< 0.001	16.33 (13.43, 19.65)	7.43 (3.23, 11.62)	*r* = 0.42	<0.001	< 0.009
	Control group(n=37)	49.07 [48.15, 50.00]	52.77 [50.46, 65.74]	Z=-5.240, *p* = 0.001	8.91 (6.34, 12.19)				
Pain and Hurt	Intervention group(n=39)	50.00 [37.50, 50.00]	50.00 [37.50, 50.00]	Z=-2.121, *p* = 0.034	1.92 (0.48, 3.98)	-0.10 (-2.42, 2.21)	*r* = 0.04	0.714	1.000
	Control group(n=37)	50.00 [50.00, 50.00]	50.00 [50.00, 50.00]	Z=-2.449, *p* = 0.014	2.03 (0.69, 3.75)				
Nausea	Intervention group(n=39)	50.00 [40.00, 50.00]	50.00 [45.00, 50.00]	Z=-2.233, *p* = 0.026	1.41 (0.44, 2.50)	0.46 (-1.03, 1.96)	*r* = 0.07	0.553	1.000
	Control group(n=37)	50.00 [45.00, 50.00]	50.00 [47.50, 60.00]	Z=-1.890, *p* = 0.059	0.95 (0.24, 1.86)				
Treatment Anxiety	Intervention group(n=39)	50.00 [50.00, 50.00]	75.00 [66.67, 75.00]	Z=-5.126, *p*< 0.001	20.51 (16.67, 24.78)	9.03 (3.42, 14.64)	*r* = 0.34	0.002	0.018
	Control group(n=37)	50.00 [50.00, 50.00]	66.67 [50.00, 70.83]	Z=-4.166, *p*< 0.001	11.49 (7.93, 15.36)				
Procedural Anxiety	Intervention group(n=39)	50.00 [33.33, 50.00]	75.00 [58.33, 75.00]	Z=-5.359, *p*< 0.001	23.08 (19.90, 26.35)	10.91 (6.06, 15.86)	*r* = 0.46	<0.001	< 0.009
	Control group(n=37)	50.00 [45.83, 50.00]	58.33 [50.00, 66.67]	Z=-4.601, *p*< 0.001	12.16 (8.96, 15.55)				
Worry	Intervention group(n=39)	50.00 [41.67, 50.00]	75.00 [50.00, 75.00]	Z=-4.826, *p*< 0.001	20.73 (15.55, 26.44)	8.79 (1.72, 15.86)	*r* = 0.28	0.014	0.126
	Control group(n=37)	50.00 [50.00, 50.00]	50.00 [50.00, 75.00]	Z=-3.853, *p*< 0.001	11.94 (7.67, 16.94)				
Cognitive Problems	Intervention group(n=39)	50.00 [40.00, 50.00]	75.00 [55.00, 75.00]	Z=-4.927, *p*< 0.001	21.28 (16.33, 26.83)	10.20 (3.47, 16.93)	*r* = 0.34	0.003	0.027
	Control group(n=37)	50.00 [50.00, 50.00]	50.00 [50.00, 75.00]	Z=-3.844, *p*< 0.001	11.08 (6.79, 16.45)				
Perceived Physical Appearance	Intervention group(n=39)	50.00 [41.67, 50.00]	75.00 [58.33, 75.00]	Z=-4.981, *p*< 0.001	21.58 (16.67, 26.98)	10.09 (3.17, 17.02)	*r* = 0.33	0.004	0.036
	Control group(n=37)	50.00 [50.00, 50.00]	50.00 [50.00, 75.00]	Z=-3.767, *p*< 0.001	11.49 (7.18, 16.72)				
Communication	Intervention group(n=39)	50.00 [41.67, 50.00]	75.00 [58.33, 75.00]	Z=-5.170, *p*< 0.001	22.01 (17.27, 27.07)	10.30 (3.49, 17.10)	*r* = 0.35	0.002	0.018
	Control group(n=37)	50.00 [50.00, 50.00]	50.00 [50.00, 75.00]	Z=-3.929, *p*< 0.001	11.71 (7.42, 17.11)				

PedsQL™ 3.0, Pediatric Quality of Life Inventory™ 3.0 Cancer Module. Values are mean ± SD or median [IQR]; Δ, within-group change; ΔΔ, between-group difference in change. Effect sizes are Cohen’s *d* or rank-biserial *r*; *p* values represent between-group comparisons; *Adj. p* =Bonferroni-adjusted p value for between-group change.

## 4 Discussion

This quasi-experimental study evaluated the effects of an eight-week group painting therapy program on psychological distress, post-traumatic growth, and health-related quality of life among children and adolescents with bone tumors. Participants in the intervention group showed greater reductions in anxiety and depressive symptoms, as well as improvements in selected domains of post-traumatic growth and health-related quality of life, compared with the control group. The most consistent effects were observed for anxiety-related outcomes, whereas improvements in depressive symptoms and post-traumatic growth were more domain-specific. Changes in quality of life were primarily evident in psychosocial domains rather than physical symptom domains. These findings suggest that structured group painting therapy may provide additional psychosocial support within pediatric bone tumor care, particularly given the moderate baseline levels of distress and relatively low post-traumatic growth and quality of life observed in this population.

Anxiety demonstrated the largest magnitude of change. Reductions in total anxiety and across subdomains were more pronounced in the intervention group, consistent with previous studies showing that art-based interventions in pediatric oncology are particularly effective in reducing anxiety (24, 37). Painting may facilitate the externalization of internal tension and treatment-related fears through symbolic expression. The focused and immersive nature of creative activity may also interrupt ruminative thinking and reduce physiological arousal (38). The group format likely contributed to emotional normalization and peer support, both of which are especially relevant during prolonged hospitalization (39, 40). Improvements in the control group may reflect natural psychological adaptation or supportive caregiving influences (41), indicating that multiple factors contribute to anxiety reduction during treatment.

Depressive symptoms also declined in the intervention group, although the magnitude of improvement was smaller than that observed for anxiety. Similar, though less robust, improvements in depressive symptoms have also been described in previous art therapy studies involving children and adolescents with cancer (42, 43). Painting may influence mood through opportunities for emotional expression, mastery experiences, and social interaction (39, 44). Secondary pathways may also be relevant, such as increased willingness to engage in rehabilitation activities or new channels of communication within families through discussion of artwork. At the same time, depressive symptoms in pediatric oncology are influenced by prolonged treatment exposure, physical limitations, family dynamics, and cultural norms surrounding emotional communication (45, 46). These contextual influences may constrain short-term improvement. Adolescence is a developmental period characterized by identity formation and heightened sensitivity to bodily change (47). Illness-related disruptions during this stage may affect emerging self-concept and perceptions of continuity and future possibility. Creative processes may support gradual reconstruction of self-perception and agency, although longer follow-up is needed to determine whether these effects are sustained.

Post-traumatic growth improved in the intervention group overall, although the effects were domain-specific and no significant between-group difference was observed for spiritual change. This pattern is consistent with theoretical models proposing that growth emerges through cognitive and emotional processing and meaning-making following adversity (48, 49). However, previous studies have not yielded entirely consistent findings. For example, a group art therapy study of patients with hematologic malignancies found no significant effect of art therapy on post-traumatic growth (50). This discrepancy may partly reflect differences in patient age and disease context, intervention duration and setting, as well as the measurement of post-traumatic growth. The reflective and symbolic elements of painting may provide a structured context for integrating illness experiences into a more coherent personal narrative. Group interaction may further support this process by facilitating shared meaning-making (40). The absence of significant change in the domain of spiritual change suggests that future-oriented dimensions of growth may require longer developmental timeframes and broader social engagement. Cultural values in China that emphasize endurance, gratitude, and family obligation may also shape children’s appraisal of experiences, underscoring the importance of multi-informant and longitudinal designs in future research (51).

Health-related quality of life improved primarily in psychosocial domains, including treatment anxiety and procedural anxiety, cognitive functioning, perceived physical appearance, and communication. The between-group effect on the worry domain did not remain statistically significant after Bonferroni correction, possibly because this dimension reflects cancer-specific concerns about treatment effectiveness, treatment-related side effects, and recurrence risk, rather than broader anxiety symptoms encountered in daily life. This pattern is consistent with previous studies suggesting that painting- or art-based interventions improve quality of life mainly in emotional and social domains rather than in physical symptom domains (52, 53). Psychological distress is closely linked to quality-of-life impairments in pediatric oncology, and reductions in emotional symptoms likely contributed to these changes. Gains in perceived physical appearance may be particularly meaningful during adolescence, when body image plays a central role in self-perception and social integration (47). Creative expression may support a sense of reclaiming agency over bodily changes and reconstructing a more integrated self-image. In contrast, physical symptom domains such as pain and nausea did not differ significantly between groups. These negative findings may reflect the fact that pain and nausea are more directly influenced by disease status, treatment intensity, and pharmacological symptom management than by psychosocial intervention alone. In this study, symptom management was consistent across both groups, with analgesics and antiemetics administered according to the same institutional protocols and individual clinical needs. Therefore, the significant improvement in the PedsQL™ 3.0 total score was likely driven primarily by improvements in the psychosocial and adaptation-related domains, rather than by differential changes in the worry or physical symptom domains. This distinction suggests that painting therapy may primarily influence psychological adaptation rather than somatic symptom burden, which requires coordinated medical management (21). Differences in baseline symptom burden, engagement with the intervention, and disease- or treatment-related factors may also have contributed to variability across quality-of-life domains.

Although feasibility was not formally evaluated, the low attrition rate and absence of reported adverse events indicate that the intervention was acceptable and safe within the inpatient setting. The control condition functioned as an active attention control (54), and structured peer interaction alone may have contributed to some improvements. However, the more consistent changes observed in the intervention group suggest benefits beyond nonspecific social contact. Given the non-randomized design and short follow-up period, findings should be interpreted cautiously. Nonetheless, the overall pattern indicates that group painting therapy may provide meaningful psychosocial support during active treatment for pediatric bone tumors.

### 4.1 Limitations

This study has several limitations. It was conducted in a single center with a relatively small sample size, which may limit external validity. Allocation was ward-based rather than randomized, and clustering effects or residual confounding cannot be fully excluded, as no covariate-adjusted or cluster-corrected analyses were performed, despite comparable baseline characteristics. All outcomes were self-reported and participants were not blinded, which may introduce reporting bias. The control group received nurse-facilitated peer communication as an active attention control, which may have attenuated between-group differences and complicated interpretation of intervention-specific effects. Outcomes were assessed only within 24 hours after completion of the program, precluding conclusions regarding long-term sustainability. Finally, treatment regimens, symptom fluctuations, individual engagement, and parental involvement were not fully controlled. Future research using randomized or cluster-adjusted designs, multi-center samples, longer follow-up periods, and multi-method assessment is needed to strengthen the evidence base and clarify mechanisms of action.

## 5 Conclusion

Group painting therapy is a structured expressive intervention that offers children and adolescents with bone tumors a developmentally appropriate setting for emotional expression, reflection, and peer connection. In this quasi-experimental study, participation was associated with reductions in anxiety and depressive symptoms, improvements in selected domains of post-traumatic growth, and enhancement of psychosocial aspects of health-related quality of life. While results should be interpreted cautiously given design limitations, the findings suggest that integrating structured group painting therapy into pediatric oncology services may represent a feasible and supportive adjunct to routine care. Further rigorous and longitudinal studies are warranted to confirm effectiveness, examine sustainability, and clarify mechanisms underlying observed benefits.

## Data Availability

The original contributions presented in the study are included in the article/supplementary material. Further inquiries can be directed to the corresponding author.
